# Network Analysis of Neurodegenerative Disease Highlights a Role of Toll-Like Receptor Signaling

**DOI:** 10.1155/2014/686505

**Published:** 2014-01-16

**Authors:** Thanh-Phuong Nguyen, Laura Caberlotto, Melissa J. Morine, Corrado Priami

**Affiliations:** ^1^The Microsoft Research, University of Trento Centre for Computational Systems Biology (COSBI), Piazza Manifattura 1, 38068 Rovereto, Italy; ^2^Department of Mathematics, University of Trento, Via Sommarive, 14-38123 Povo, Italy

## Abstract

Despite significant advances in the study of the molecular mechanisms altered in the development and progression of neurodegenerative diseases (NDs), the etiology is still enigmatic and the distinctions between diseases are not always entirely clear. We present an efficient computational method based on protein-protein interaction network (PPI) to model the functional network of NDs. The aim of this work is fourfold: (i) reconstruction of a PPI network relating to the NDs, (ii) construction of an association network between diseases based on proximity in the disease PPI network, (iii) quantification of disease associations, and (iv) inference of potential molecular mechanism involved in the diseases. The functional links of diseases not only showed overlap with the traditional classification in clinical settings, but also offered new insight into connections between diseases with limited clinical overlap. To gain an expanded view of the molecular mechanisms involved in NDs, both direct and indirect connector proteins were investigated. The method uncovered molecular relationships that are in common apparently distinct diseases and provided important insight into the molecular networks implicated in disease pathogenesis. In particular, the current analysis highlighted the Toll-like receptor signaling pathway as a potential candidate pathway to be targeted by therapy in neurodegeneration.

## 1. Introduction 

Neurodegenerative diseases (NDs) represent a large group of neurological disorders with heterogeneous clinical and pathological traits that are characterized by progressive nervous system dysfunction. These disorders are often associated with atrophy of the affected central or peripheral structures of the nervous system and they arise for unknown reasons and progress in a relentless manner.

Neurodegenerative disorders are a major focus of scientific and clinical interest due to their prevalence, complex biochemistry and pathology, and lack of mechanism-based treatments. Their number is currently estimated to be a few hundred, and, among these, many appear to overlap with one another clinically and pathologically, rendering their practical classification quite challenging. The most popular categorization of neurodegenerative disorders is still based on the predominant clinical feature or the topography of the predominant lesion or often on a combination of both [1], but since the associated etiology and neuropathology are still unknown, there are limitations of the current framework of neurodegenerative diseases.

The recent expansion of public interactomics databases allows researchers to advance computational methods for network medicine [2]. Network medicine aims to explore the pathogenic mechanism of a particular disease, and additionally to infer the complex associations of diseases in a systematic point of view. One of the major approaches is the exploration of the human protein-protein interaction (PPI) network to study disease genes via their corresponding product proteins (disease proteins), which are then used to construct the disease PPI network [3]. Disease research based on PPI network has achieved noteworthy results [4–9]. Among them, some recent studies have analyzed NDs using PPI; however, they mostly considered a specific disease, such as Alzheimer's disease [10–12]. Another work inferred overlapping regulators of NDs in different organisms [13], the direct commonality among NDs in term of pathways [14], or the reconstruction of the NDs network based on PPI networks, regulatory networks, and Boolean networks [15]. The previous work mostly concentrated on constructing the PPI network related to NDs but has not yet quantified the topological associations among NDs. Moreover, the indirect network relationships underlying functionality associations between NDs have not been clarified yet.

We present an efficient computational method based on PPI network for studying NDs. We selected nine NDs based on their prevalence and/or on the relevance for the different molecular, genetic, or clinical aspects of these complex disorders: Huntington's disease (HD), prion (P), frontotemporal dementia (FTD), Alzheimer's disease (AD), Friedreich's ataxia (FA), Lewy body disease (LBD), Parkinson's disease (PD), amyotrophic lateral sclerosis (ALS), and spinal muscular atrophy (SMA). Clinically, these degenerative disorders of the brain are characterized by marked loss of memory (AD, FTD, LBD, and prion), movement disorders (HD, FTD, LBD, PD, and SMA), and weakness or poor balance (ALS, FTD, prion, FA).

In addition to the nine NDs, glioblastoma multiforme (GBM); a cancer affecting the central nervous system (CNS) was considered to investigate the effects of a disease not related to neurodegeneration in the ND network perturbation. GBM is the most common and most aggressive malignant primary brain tumor in humans, involving glial cells and accounting for the majority of all functional tissue brain tumor cases.

We first reconstructed the network of disease proteins related to the ten diseases of interest. We then identified key nodes of significant influence using topological network indices, namely, degree, betweenness, closeness, and topological importance. The highly ranked proteins were found and biologically interpreted. Shortest paths between pairs of disease proteins were computed to measure the disease linkages and to identify sharing proteins and connecting proteins, most likely responsible for disease linkages. We modeled the network of diseases by computing different metrics using a single path length and combination of various path lengths. The relatedness between diseases in the network supported the traditional clinical classification of the diseases but uncovered also relationships among disease pairs that classically are not so strongly related. The sharing and connecting proteins were evaluated and the results showed their significant functionality in the pathogenesis of NDs. Toll-like receptor (TLR) signaling pathway was highlighted to be a prominent mechanism in the overall connector proteins providing potential candidate proteins to be targeted by pharmacological intervention (e.g., TRAF6).

We then applied text mining proposed in [16] to reconstruct the network of NDs, as a means to complement the network analysis with a literature-based approach. The text mining method was seemingly biased because the major data sources were curated literature in both the OMIM database [17] and the MeSH database [18]. Our proposed method was based on protein interactions, which are defined independently from previous clinical classifications. Moreover, while text mining method could reveal only the phenotype similarity, our method quantitatively measured the disease relatedness based on the network topology and studied more comprehensively the functional mechanism of linkages in terms of connector proteins.

In the present study, the interaction network of NDs was studied to uncover the direct and indirect molecular mechanisms underlying the connections between NDs. It is expected that the characterization of a disease protein interaction network could potentially uncover molecular relationships that are in common between apparently distinct diseases and provide important insight into the molecular networks implicated in disease pathogenesis.

## 2. Materials and Methods

### 2.1. Materials

We investigated two main databases: a disease phenotype database as the Online Mendelian Inheritance in Man (OMIM) database [17] and a protein interaction database as the Interologous Interaction Database (i2d) database [19]. The set of ND genes was curated from the OMIM database, which is a catalogue of human genes and genetic disorders. In OMIM, the list of hereditary disease genes is described in the OMIM morbid map. The protein interaction network was constructed based on the i2d database, which is an integrated database of almost all known experimental and predicted human protein interaction data sets (including HRPD, BIND, and BioGrid). The comprehensive interaction data published in the i2d database enabled our analysis on a complete network of disease proteins. Identifiers of proteins were unified using the protein IDs defined in the Uniprot database [20]. Our study considered the database versions released in August 2011.

### 2.2. Methods

**Figure 1:**
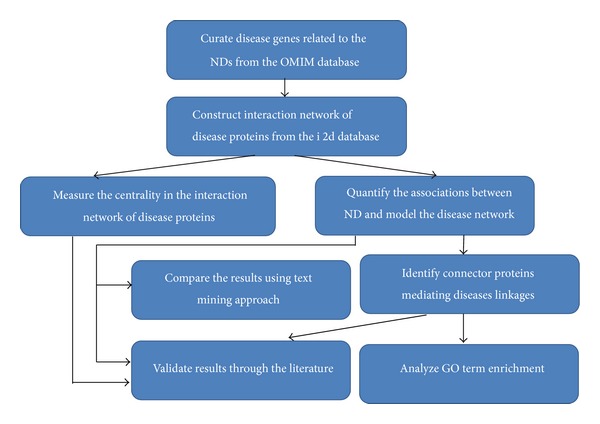
The framework of studying functional interactions network of NDs.

The framework of the analysis is shown in Figure 1. Based on the set of curated ND genes, we first identified the network of disease proteins related to the ten diseases of interest. To assess the topological features of the proteins, we measured several centralities in the network, namely, degree, betweenness, closeness, and topological importance. The network of diseases was later modeled by calculating disease linkages in terms of shortest paths between pairs of disease proteins. We identified sharing proteins and connecting proteins as the main components that were most likely responsible for disease linkages. The functionality of connecting proteins was studied by the GO enrichment analysis. The following sections present the framework in details.

### 2.3. Modeling Interaction Network of Disease Proteins

To investigate the NDs from the network point of view, we first modeled the interaction network of diseases proteins. Disease proteins are product proteins of disease genes that are related to specific neurodegenerative disorders. From the morbid map published in the OMIM database, we obtained the list of disease genes related to the 10 diseases. In order to construct the protein interaction network related to the 10 diseases, we mapped the disease genes to disease proteins based on the mapping scheme of the Uniprot database. The interactions of those disease proteins were then obtained by exploring the experimentally validated interactions from the i2d database. All the homologous predicted protein interactions in the i2d database were excluded to increase the reliability of protein interaction data. The final interaction network of interest contained the disease proteins (nodes) and their direct interacting partners (edges). We took into account only direct interactions (i.e., first degree neighbors). The network was undirected and weighted because we considered the binary interactions.

### 2.4. Modeling Interaction Network of NDs

Based on the constructed network of the disease proteins, we modeled the network of NDs with metanodes and metaedges that represented the diseases and the connections between diseases, correspondingly. A meta-node was defined as a cluster of the disease proteins related to one disease. A meta-edge connecting from one disease to the other was defined as a cluster of the paths between their disease proteins. The meta-edges were weighted using different score functions *r*_*i*_ to demonstrate the strength of the association between two diseases. For example, the two meta-nodes, named ALS and SMA, consisted of all the proteins related to ALS and SMA correspondingly. For each pair of proteins (one belonging to ALS, one belonging to SMA), we computed paths connecting them and then grouped all of the paths together to identify a meta-edge, named ALS-SMA (undirected edge) with weight calculated by *r*_*i*_. The meta-edges are visible if the score *r*_*i*_ > 0.

### 2.5. Analyzing the ND Network Using the Network Mining Approach

#### 2.5.1. Computing Topological Properties of Protein Interaction Network

To understand networks and their participating proteins, we evaluated the centrality of proteins in the network. The functional importance of proteins might be inferred from their central roles in the network [21–24]. Since each centrality describes a unique structural feature, reliable predictions of the biological properties can be achieved by combinations of these measures, rather than relying on a single index. We computed a number of centralities varying from local scale (degree and eigenvector scores) to intermediate scale (topological importance up to 1 and 3 steps) and finally to global scale (betweenness and closeness).

A number of centralities have been used to characterize the networks studied as follows:

(i) The first, the degree centrality or connectivity (*D*) of a protein *v*_*i*_, indicates how many interactions *e*_*ij*_ the protein has to the other proteins *v*_*j*_. This is the most popular to evaluate the local centrality in the network [25].
(ii) The second, betweenness centrality (*B*), is a measure of the positional influence of proteins in the networks. The betweenness centrality of a protein *v*_*i*_ is defined as the number of shortest paths *p* between pairs of other proteins that run through *v*_*i*_ over the total number of shortest paths between pairs of other proteins [25].
(iii) The third, the closeness centrality (*C*), measures how close a protein is to others. The farness of a node *v*_*i*_ is defined as the sum of its distances to all other nodes, and its closeness is defined as the inverse of the farness. Closeness considers the distance of the path than the number of the path like *B* does. A protein with high *C* easily catches other proteins in a short time [25].
(iv) The fourth, the topological importance (TI^*n*^) index, quantifies indirect interactions of various lengths *n* separately. It is based on the relative number of interactions connecting one protein to its surrounding proteins, under the consideration of the whole arrangement of interactions (direct or indirect) among those satellite proteins [26].

#### 2.5.2. Computing the Association Scores between NDs

We carried out three steps to compute the association scores between NDs by using the shortest path information.

Given two ND diseases, A and B, the procedure was presented as follows.

**Step 1**

Find all the shortest paths *p*_*k*_ between *v*_*i*_ and *v*_*j*_, for all protein pairs (*v*_*i*_, *v*_*j*_), where *v*_*i*_ is a protein related to disease A, *v*_*j*_ is a protein related to disease B.

**Step 2**

Calculate the length/distance *l*_*k*_ of the path *p*_*k*_.

**Step 3**

Compute the score between A and B based on single path length metric *r*_1_ or combined path length metrics, *r*_2_ and *r*_3_.

**Figure 2:**
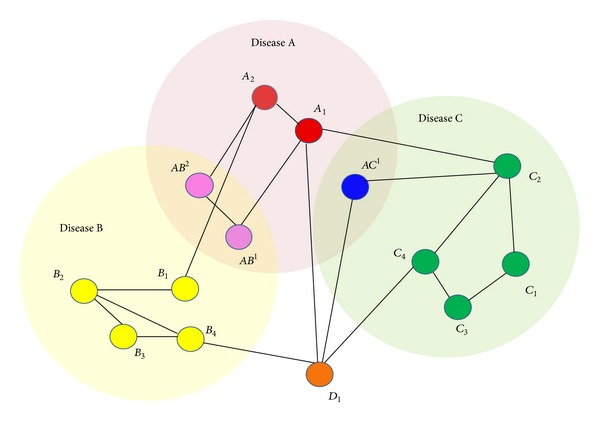
A toy model for analyzing the ND network based on shortest paths. An illustration showing how the shortest path and scores discussed in the report are calculated. Red, yellow, and green proteins are involved in three diseases A, B, and C correspondingly. Pink proteins *AB* ^1^, and *AB* ^2^ are shared between the two diseases A and B, while a blue protein *AC* ^1^ is shared between the two diseases A and C. Considering protein *A*_1_  relating disease A as start protein and proteins *C*_2_,  *C*_3_,  *C*_4_ relating disease C as end proteins, there are *p*_1_ = (*A*_1_ → *C*_2_) and *l* = 1;  *p*_2_ = (*A*_1_ → *D*_1_ → *C*_4_ → *C*_3_)  and *l* = 3;  *p*_3_ = (*A*_1_ → *D*_1_ → *C*_4_) and *l* = 2. The orange protein *D*_1_ is a connector protein mediating the connection between two diseases B and C (*B*_3_ → *D*_1_ → *C*_4_).

The shortest path problem is to find a path *p*_*k*_ having the minimal path length. A Breadth-First Search algorithm [27, 28] has been employed to find the shortest paths between two proteins (the start protein *v*_*i*_ and the end protein *v*_*j*_). The shortest paths may have different path lengths (*l*_*k*_ = 0,  *l*_*k*_ = 1,  *l*_*k*_ = 2,  *l*_*k*_ = 3, etc.). In the example shown in Figure 2, there are different shortest paths from start protein *A*_1_ related to disease A to end proteins *C*_2_,  *C*_3_,  *C*_4_ related to disease C, with *l*_*k*_ = 1 (*A*_1_ → *C*_2_), *l*_*k*_ = 3 (*A*_1_ → *D*_1_ → *C*_4_ → *C*_3_), and *l*_*k*_ = 2 (*A*_1_ → *D*_1_ → *C*_4_) correspondingly. Here we defined connector proteins as proteins visited along the path except the start protein and the end protein. We note that in this work we considered the one-step connector proteins only because this work focused on close links between diseases. The larger set of connector proteins has been under investigation and would be analyzed in future work.

If the path length *l*_*k*_ equals to 0, this demonstrates the occurrence of one common protein at least between two NDs, for example, protein *AC* ^1^ between diseases A and C or proteins *AB* ^1^, and *AB* ^2^ between diseases A and B. If the path length *l*_*k*_ equals 1, this signifies a direct connection, for example, two proteins *A*_1_ and *C*_2_ are directly connected. If the path length *l*_*k*_ equals to 2, the path consists of three nodes: a start protein (*A*_1_), a connector protein (*D*_1_), and an end protein (*C*_4_). Using this form of analysis the path lengths were used to obtain the scores between two NDs.

For analyzing how close two NDs are, we proposed three metrics based on the path length, *r*_1_, *r*_2_, and *r*_3_.

(i) Score *r*_1_ (*D*_1_, *D*_2_) is defined as the number of paths *n*_*k*_ between two disease metanodes by considering separately three path lengths *l*_*k*_ = 0, *l*_*k*_ = 1, and *l*_*k*_ = 2. The score *r*_1_ shows how two diseases are related in different network distances. Given a specific path length, two diseases are more related if they have more paths. For example, in Figure 2, when considering *l*_*k*_ = 0, two diseases A and B being interlinked by two paths and two diseases A and C being interlinked by one path, then *r*_1_ (A, B)>*r*_1_ (A, C). As a result, disease B has probably a closer association to disease A than disease C does.
(ii) Score *r*_2_ (*D*_1_, *D*_2_) is defined as the combination of the three most significant paths with length *l*_*k*_ = 0, *l*_*k*_ = 1, and *l*_*k*_ = 2. Consider
  (1) $$\begin{array}{c} r_{2}=2*n_{l=0}+1*n_{l=1}+\frac{1}{2}*n_{l=2}, \end{array}$$
 where *n*_*l*=0_,  *n*_*l*=1_, and *n*_*l*=2_ are the number of paths with *l*_*k*_ = 0,  *l*_*k*_ = 1, and *l*_*k*_ = 2, correspondingly.
(iii) Score *r*_3_ (*D*_1_, *D*_2_) is defined as the combination of all of the found. Consider
  (2) $$\begin{array}{c} r_{3}\left(D_{1},D_{2}\right)=\delta_{0}+\left(1-\sum_{i=1}^{L}\frac{1}{l_{i}}*n_{i}\right), \end{array}$$
 where
  (3) $$\begin{array}{c} \delta_{0}=\{\begin{array}{cc} 1 & \text{if}\;n_{0}\neq 1(\text{there}\;\text{is}\;\text{at}\;\text{least}\;\text{one}\;\text{common}\;\\ & \;\;\;\;\;\text{protein}\;\text{between}\;\text{two}\;\text{diseases}),\\ 0 & \text{otherwise}; \end{array} \end{array}$$
 
*n*_*i*_: the number of paths with length *l*_1_ ( = 1), *l*_2_ ( = 2),…, *l*_*L*_ ( = *L*), correspondingly;
 
*L*: the maximum length corresponding to the furthest path found.

The network analysis was implemented in CoSBiLab-Graph [29] and Centralities in Biological Networks [30]. The network mining and text mining were performed by Python script [31]. The network visualization was performed by the software NAViGaTOR (Network Analysis, Visualization, and Graphing Toronto) [32].

### 2.6. Analyzing the ND Network Using Text Mining Approach

Here we used phenotype similarity data developed by van Driel [16]. Their work mined the textual data from the OMIM database and the hierarchical structure of the anatomy and the disease sections of the medical subject headings vocabulary (MeSH) [18] and calculated the phenotype similarity. The pairwise matrix was produced for over 5000 disease phenotypes. We mined the OMIM database and obtained all of the phenotypic terms associated to the ten diseases of interest. The submatrix of those terms was generated to calculate the correlation between the NDs. The weights of meta-edges in the disease network were assigned by using the correlation values in the submatrix.

### 2.7. GO Term Enrichment Analysis

We used the Cytoscape plug-in ClueGO [33] to identify gene ontology (GO) terms (from level 3 to level 8 of the GO biological process hierarchy) that were significantly enriched with the complete set of connector proteins and the connector proteins of two diseases pairs ALS-PD and FTD-PD. To increase specificity of results, only GO terms containing at least 10 connector proteins and with at least 10% coverage of the term by connector proteins were considered. A one-sided hypergeometric test was used to determine significantly enriched GO terms; *P*-values were corrected using the Benjamini and Hochberg method, and those terms with corrected *P*-value < 0.1 were considered significant [34]. ClueGO requires selection of a minimum threshold for the kappa score, which measures the association strength between overlapping GO terms (see [31] for details on the kappa index). For this analysis we used a threshold of 0.3 (i.e., GO term pairs with kappa score ≥ 0.3 are connected in the ClueGO network). All other default parameters were used.

## 3. Results

### 3.1. Interaction Network of ND Proteins

We obtained 75 disease proteins based on the extracted data from the OMIM database. Among the 75 disease proteins, 71 have interactions published in the i2d database. The complete list of disease proteins and their corresponding diseases are presented in additional file 1: Suppl.1 (see Supplementary Material available online at http://dx.doi.org/10.1155/2014/686505). From the set of the 71 disease proteins, we constructed the interaction network of proteins related to the ten diseases. The network consisted of 1,222 proteins and 1,521 interactions. The network is not fullyconnected, having six separated components (or six subnetworks). The largest component contained 1,198 proteins and 1,502 interactions, corresponding to the giant component of the network (the one for which we mainly carried out network analysis).  Table 1 shows the statistics of the six components and the list of proteins corresponding to the six components is provided in the additional file 1: Suppl.2.

A number of indices were calculated for highlighting the key proteins in the network. The top 20 proteins ranked by the four indices, that is, *D*,  *B*,  *C*, and TI^3^, are shown in Table 2. The ranks using *D*,  *B*, and TI^3^ were almost identical. The most central proteins are v-erb-b2 erythroblastic leukemia viral oncogene homolog 2, neuroglioblastoma derived oncogene homolog (avian), TATA box binding protein, and prion protein.

Tv-erb-b2 erythroblastic leukemia viral oncogene homolog 2 (Uniprot ID: P04626), the highest-ranked protein, is a member of the epidermal growth factor (EGF) receptor family of receptor tyrosine kinases. ERBB2 is considered an oncogene and it promotes cellular growth and survival [35]. This protein has no ligand binding domain of its own, but it does bind tightly to other ligand-bound EGF receptor family members to form a heterodimer [36]. It is an essential component of a neuregulin-receptor complex, although neuregulins do not interact with it alone [37]. Although ERBB2 has been strongly associated to cancer, several recent findings suggest a role of neuregulin signaling in synaptic maintenance and possibly neurodegenerative diseases [38]; thus, being the highest ranked key node in the network could support the hypothesized strong involvement of this protein not only in cancer, but also in neurodegeneration.

Although the *closeness*-rank orders were not totally overlapping, the list of ten most central proteins was conserved across most centrality indices. The highest ranked proteins are microtubule-associated protein tau, growth factor receptor-bound protein 2 (UniprotID: P10636), and synuclein, alpha (non-A4 component of amyloid precursor) (UniprotID: P62993). Those proteins may not have many direct neighbors (measured by *D*) or have many paths crossing through (measured by *B*), but they are likely to be close to numerous proteins due to their short-distance paths. Among those, microtubule-associated protein tau (MAPT) promotes microtubule assembly and stability and might be involved in the establishment and maintenance of neuronal polarity [39]. Aggregates of hyperphosphorylated forms of tau protein participate in the formation of neurofibrillary tangles, which characterize numerous neurodegenerative disorders named tauopathies. Tau pathology represents a primary pathogenic event in various neurodegenerative diseases [40]. More than 40 mutations in the MAPT gene have been also found to cause frontotemporal dementia with parkinsonism-17 (FTDP-17) [41]. Being one of the highest ranked proteins in this CNS disease network, it could play a key role not only in tauopathies physiopathology, but also in other neurodegenerative disorders. Pathogenic roles of other highly ranked proteins are further addressed in Section 4.

**Figure 3:**
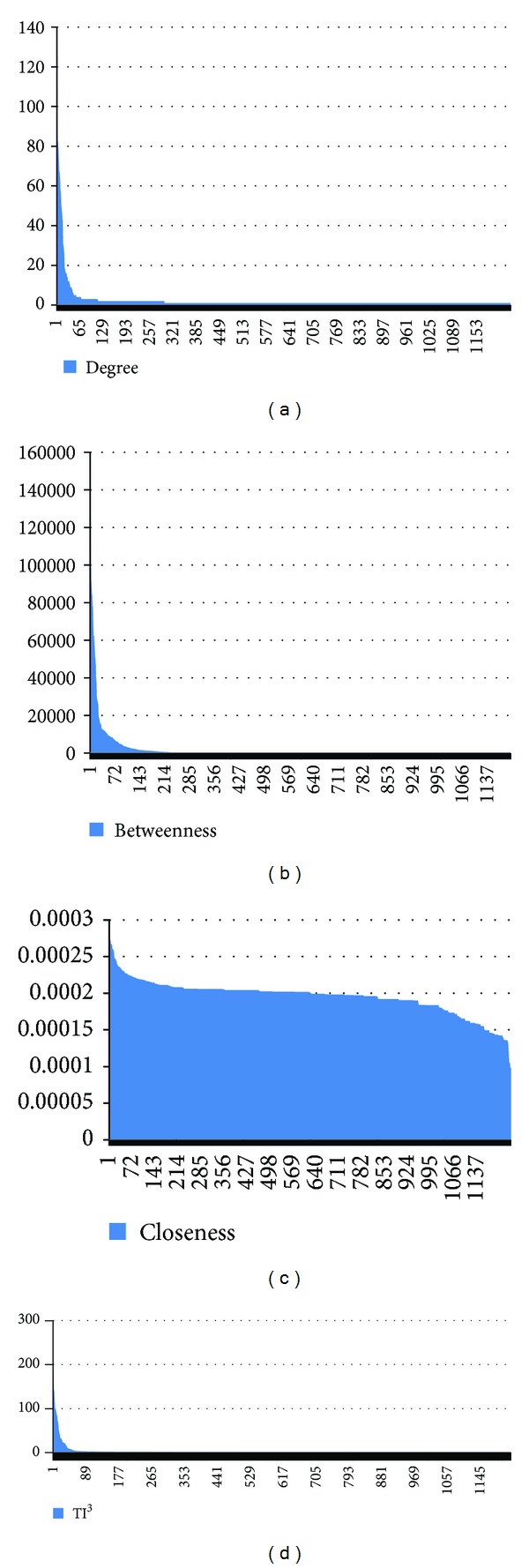
The distribution diagrams of the four network indices calculated for the network of disease proteins.

Figure 3 shows the distribution diagrams of the four indices. The three distributions of the indices *D*,  *B*, and TI^3^ follow a strongly left-skewed distribution except the unimodal, normal-like distribution of the *C* index. The ranking tables and distribution diagrams for the three indices are reported in additional file 1: Suppl.3.

### 3.2. Interaction Network of Diseases Based on Network Mining

**Figure 4:**
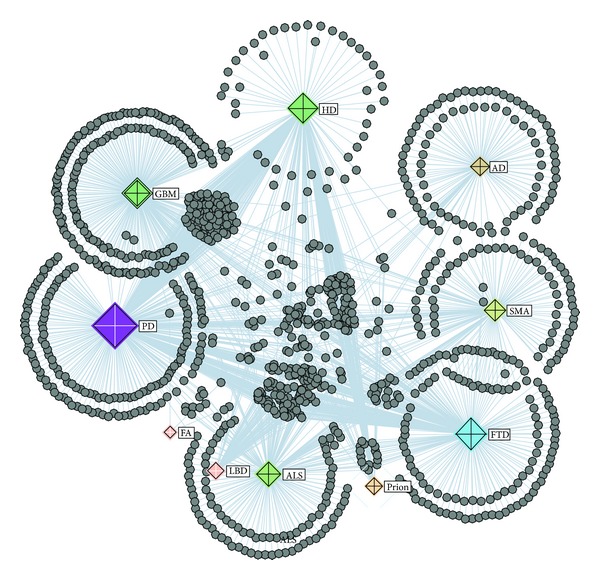
Protein-protein interaction network with collapsed 10 meta-nodes. Disease nodes are the diamond nodes and proteins interacting with disease proteins are the circular nodes.

The interaction network of NDs was investigated for studying the association between NDs. The network of NDs contains 10 meta-nodes representing the 10 diseases, that is, HD, P, FTD, AD, ALS, FA, LBD, PD, SMA, and GBM (see more in Section 2.2).Figure 4 shows the disease network in protein interaction point of view. In Figure 4, the 10 diseases are the diamond meta-nodes and proteins interacting with disease proteins are the circular nodes. The connections between disease nodes were identified by the use of protein interactions, for example, a strong connection between FTD and PD detected by their large cluster of protein interactions. Investigating the links between diseases could provide newer insights into diseases pathogenesis [2].

The shortest paths were computed to measure the relatedness of each pair of diseases. For instance, we identified 953 paths between ALS-PD, 753 paths between AD-PD, and 730 paths between FTD-PD. The longest paths computed are of 8-step length. We investigated the three most important path lengths *l* = 0 (with common proteins), *l* = 1 (with direct interactions) and *l* = 2 (with indirect interactions mediated by connector proteins).

It is assumed that if two diseases have a common protein (*l* = 0), they are pathogenically related to each other [2]. We found 9 proteins shared among 8 pairs of NDs, that is, PD-HD, HD-P, FTD-PD, FTD-ALS, PD-LBD, FTD-LBD, ALS-PD, and ALS-SMA. These 8 pairs of NDs are likely to have strong mutual relationships. Among them, two proteins, synuclein alpha gene and synuclein beta gene, were found in FTD, PD, and LBD. Two proteins, transient receptor potential cation channel, subfamily M, member 7, and Parkinson disease gene, autosomal recessive, early onset, were found to be shared among FTD, PD, and ALS. The list of shared proteins is presented in Table 3. In some case, those common proteins are well known for their relationships to a specific disease, such as prion protein, but their relevance to other diseases has not been discovered yet. The biological significance of the sharing proteins is discussed in Section 4.

We found 27 direct connections (*l* = 1), 24 of which belonged to the 8 pairs of NDs consisting of shared proteins. These results confirm that those diseases are likely to be strongly associated. The other three pairs of NDs with a direct link were PD-SMA, HD-GBM, and P-GBM. It is of interest that GBM has direct interactions with HD and P even though the network regulating neurodegeneration was well connected. As a result, the pathogenic links between GBM and neurodegenerative diseases could warrant further investigation.

A number of 1-step connections (*l* = 2) were computed to further explore the indirect connections between diseases. Those links require at least one connector protein for mediating the linkage between diseases. Totally, we obtained 714 indirect connections, which covered almost all of the ND pairs, except FTD-FA, AD-FA, FA-SMA, HD-FA, P-FA, and FA-LBD. The results suggested that the network mining could reveal the indirect association between the NDs. Moreover, it is known that “the network-neighbor of a disease gene is likely to cause the same or a similar disease” [4–7, 42]. The connector proteins are putative disease proteins implying pleiotropic effects.

**Figure 5:**
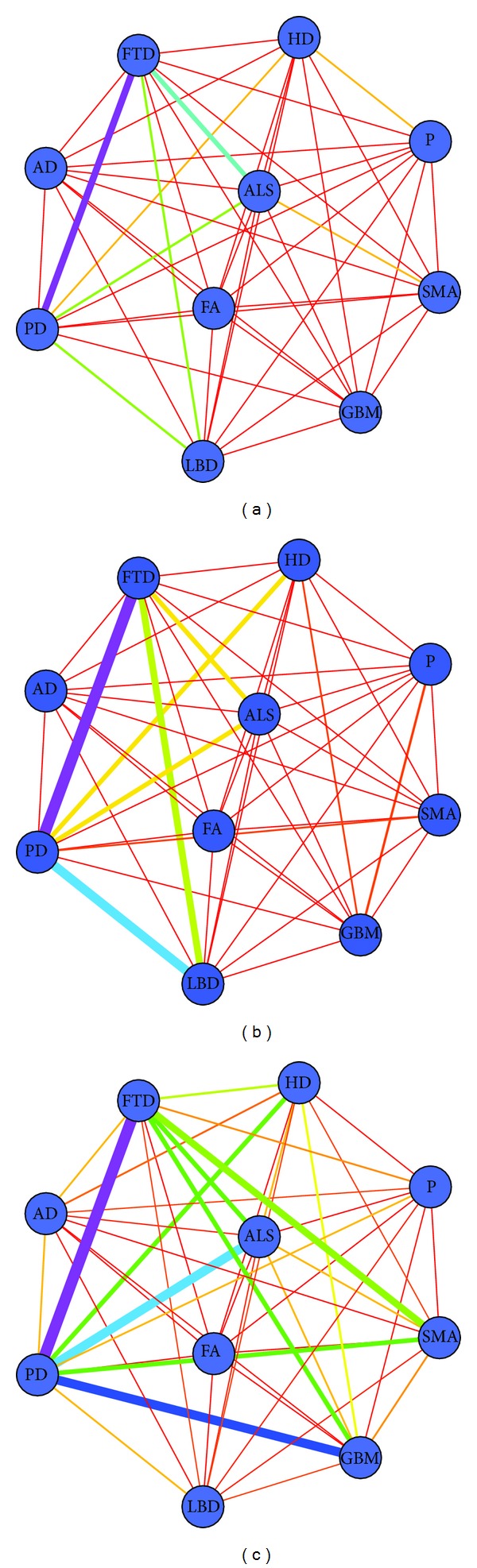
The disease network constructed using single path length metric *r*_1_. Nodes represent diseases; edges are the connections between two diseases. The thickness of the meta-edge reflects the score value *r*_1_; the stronger connection has a thicker line. (a) The disease network constructed using single path length metric *l* = 0. (b) The disease network constructed using single path length metric *l* = 1. (c) The disease network constructed using single path length metric *l* = 2.

Based on shortest path computation, we then modeled the disease network in a weighted graph of meta-nodes and meta-edges (see more in Section 2.2). We first considered the three path lengths separately by calculating *r*_1_.  Figure 5 shows the networks of NDs constructed by using the single path length, that is, *l* = 0, *l* = 1, and *l* = 2. When considering the length *l* = 0, the connection between FTD-PD was the strongest having 5 proteins in common, FTD-ALS ranked second having 3 proteins in common, and LBD-PD and ALS-PD were in the third order having 2 proteins in common. When considering the length *l* = 1, the FTD-PD connection remained the closest (with 9 direct interactions); however, LBD-PD and ALS-PD (with 6 and 3 direct interactions correspondingly) became more significant. When considering the length *l* = 2, two pairs of NDs, ALS-PD and HD-PD, were more visible owing to the number of 57 and 41 connections correspondingly.

**Figure 6:**
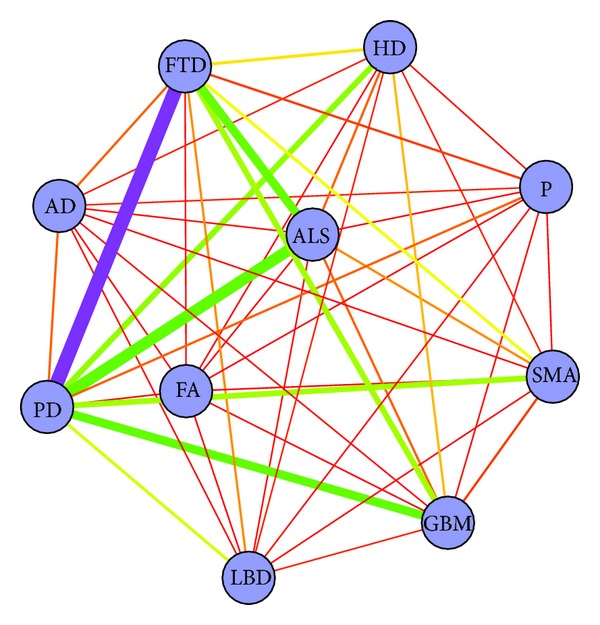
The disease network constructed using combined path length metric *r*_2_. Nodes represent diseases; edges are the connections between two diseases. The thickness of the meta-edge reflects the *r*_2_ value; the stronger connection has a thicker line.

It is interesting to combine the above three length measures to find out how the NDs connect to each other both directly and indirectly. By calculating the score *r*_2_, the network was illustrated in Figure 6. Two meta-edges FTD-PD and ALS-PD were highlighted because they obtained the highest score *r*_2_. For FTD and PD, this is probably due to the fact that there is an autosomal dominant disorder, Frontotemporal dementia and parkinsonism linked to chromosome 17 (FTDP-17), which displays clinical features in common between the two diseases [43]. ALS and PD obtained a high score possibly because they belong to a common group of movement disturbances.

**Figure 7:**
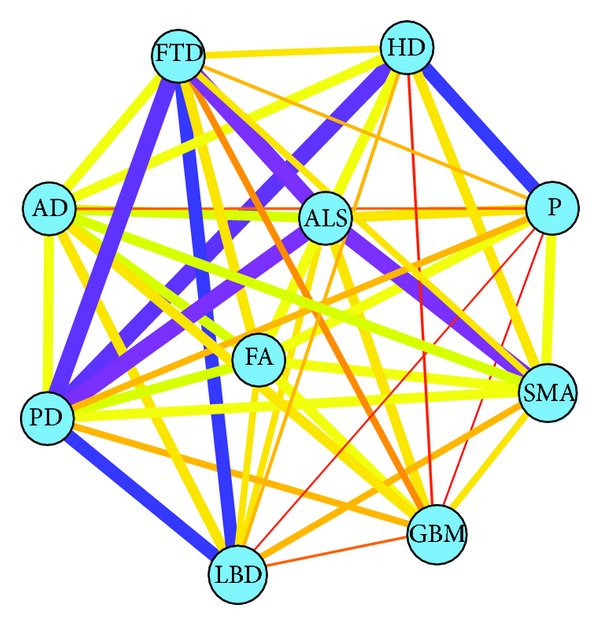
The disease network constructed using combined path length metric *r*_3_. Nodes represent diseases; edges are the connections between two diseases. The thickness of the meta-edge reflects the *r*_3_ value; the stronger connection has a thicker line.

Finally, we were interested in investigating all paths found with the wide range of lengths. Based on the score *r*_3_, we constructed the network to observe the effect of the complete set of the paths; see Figure 7. We found that the three of the four strongest connections are ALS-SMA, FTD-ALS, and HD-PD.

Our results confirm the connection between ALS-SMA, motor neuron disorders with the cardinal feature in the loss of spinal cord neurons. Although they differ in the disease development, our findings could suggest a commonality not only in the anatomical localization of the neurodegenerative process, but also in some molecular pathogenic pathways. HD and PD are related to movement disturbances, and both involve neurodegeneration in the basal ganglia; however, PD is characterized by hypokinesis and HD by hyperkinesis. Therefore, further studies are needed to uncover the potential hidden molecular alterations common to both diseases. Regarding the link between FTD-ALS, recent studies have allowed a better understanding of the overlapping spectrum of ALS and FTD, both from the clinical and the molecular point of view with a protein called TDP-43 found in the damaged tissues of both diseases [44]. Thus, our finding could strengthen this hypothesis, supported also by the clinical evidence that many people with FTD have motor neuron disease and ALS patients have subtle cognitive impairment resembling FTD.

### 3.3. Interaction Network of NDs Based on Text Mining

**Figure 8:**
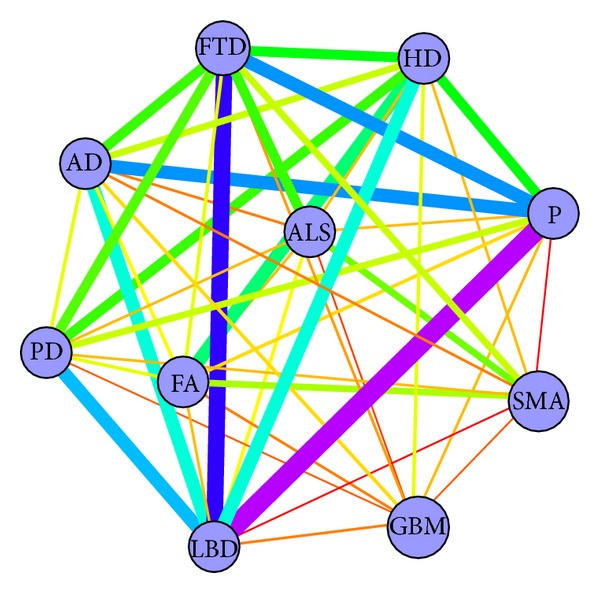
The disease network constructed using text mining. Nodes represent diseases; edges are the connections between two diseases. The thickness of the meta-edge reflects the phenotype similarity value; the stronger connection has a thicker line.

To enhance the understanding of the ND network, we used an alternative approach to infer the network (see Figure 8). In this case, the weights of the meta-edges represent the phenotype similarities. The diseases characterized clinically with dementia (FTD, AD, LBD, prion) were properly found to be highly linked. Compared to the network obtained by network mining, some connections are more evident (e.g., FTD-LBD, LBD-PD), but some of them are less visible (e.g., FTD-PD, ALS-PD). In Section 4, we will discuss the FTD-PD and ALS-PD disease pairs, suggesting the potential of the network approach to uncover the link between diseases that even with text mining approaches are unrelated.

### 3.4. GO Term Enrichment Analysis

**Figure 9:**
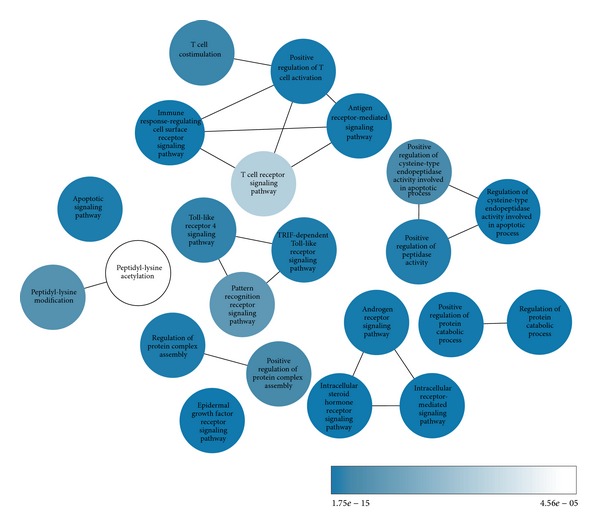
Network of significantly enriched GO terms. This schematic network illustrates GO terms that were significantly enriched in the ND connector proteins, as well as the overlap between related terms. Node colors and inset bar indicate the *P* value for enrichment of each term, after correction for multiple testing.

Figure 9 illustrates the GO terms that were significantly enriched with the ND connector proteins (*P* < 4.56*e* − 05, after correction for multiple testing). The ClueGO algorithm identified four primary functional groups among the significantly enriched terms. Two of these groups were predominantly composed of inflammatory processes, including T cell receptor signaling, T cell costimulation, TRIF-dependent TLR signaling, and TLR 4 signaling. Protein structural regulation was also overrepresented, including regulation of protein complex assembly and regulation of protein catabolic process. Finally, apoptotic processes were among the enriched GO terms, including regulation of cysteine-type endopeptidase activity involved in apoptotic process and the apoptotic signaling pathway.

## 4. Discussion

### 4.1. Direct Pleiotropic Linkage between NDs

Network analysis has become a very powerful tool to investigate not only a specific disease-relevant gene, but also to provide hypotheses on the common pathological mechanism of disorders that are currently classified as separate maladies, improving the understanding of the disease etiology and thus, possibly leading to the development of better treatments. In the present study, the associations between different neurodegenerative diseases have been explored to try to shed light on the common molecular causation or the biological pathways involved in diseases with distinct clinical features and, possibly, in this way help the clinical characterization. Different pairs of diseases seem to share specific proteins and most of the sharing node proteins found are confirming the known clinical or histopathological association between diseases. For example, the sharing node proteins for PD and LBD are alpha-synuclein (Uniprot ID: P37840) and B synuclein (UniprotID: Q16143), belonging to a family of proteins that aggregates abnormally in Parkinson's disease and Lewy body disease [45]. In fact, both pathologies could be classified as synucleinopathies.

On the contrary, among the PD and HD shared proteins, the prion protein (PrP) (Uniprot ID: P04156), surprisingly, is one of the proteins in common in these two diseases which, apart from a general motor disturbance phenotype, do not share many clinical symptoms. This protein is active in the brain and several other tissues, and although the precise function of PrP is unknown, it is likely involved in the transport of charged copper atoms (copper ions) into cells. Researchers have also proposed roles for PrP in cell signaling, cell protection, and the formation of synapses [46]. Prion gene is known to be associated with genetic prion diseases which include also Huntington disease-like syndrome [47]. Of greater interest is a recent finding of Lewy pathology in implanted embryonic dopamine neurons in PD patients, raising the intriguing possibility that PD might be a prion disorder [48]. This is in line with the hypothesis that all the diseases associated to protein misfolding are prion-related, which is also potentially supported by our findings.

### 4.2. Indirect Pleiotropic Linkage between NDs

Although the shared proteins could provide clues to identify biochemical pathways that are central to two diseases and potentially suggest some shared pharmacological treatment, of greater interest are the connector proteins which could help in shedding light on the hidden common pathophysiological mechanisms of diseases with limited clinical and pathological overlaps, especially in the field of neurodegenerative disease where the etiology is still unknown and the classification remains sometimes challenging.

**Figure 10:**
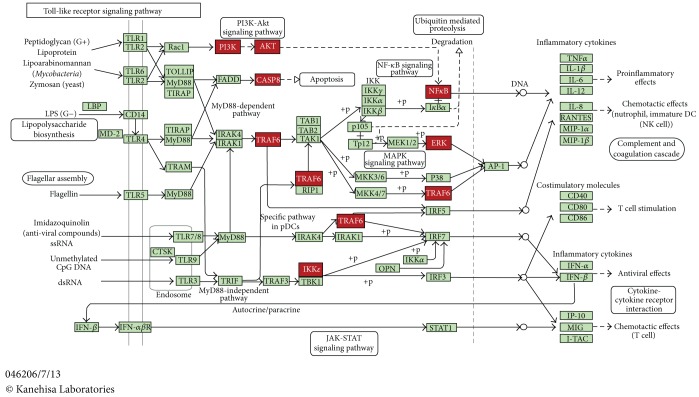
Flow diagram representing the molecular interactions in the Toll-like receptor signaling pathway (from KEGG database). The Toll-like receptors (TLRs) pathway is enriched with connector proteins, labeled in red. TLRs act as primary sensors that detect a wide variety of microbial components and elicit innate immune responses. The connector proteins found in our study (TRAF6, NF*κ*B, PI3K-AKT, ERK, IKK*ε*, and CASP8) are mediators in the TLR signaling pathway which control the expression of an array of inflammatory cytokine genes.

In additional file 1: Suppl.4, the list of connector proteins of the different diseases is presented and, interestingly, one of the most represented functional GO annotations is related to TRIF-dependent TLR signaling pathway.  Figure 10 shows the molecular interactions in the TLR signaling pathway (from KEGG database), highlighted with the mediator proteins using the DAVID tool [49]. This pathway is central to the innate immune system and the mediator proteins are related mainly to the induction of proinflammatory cytokines. However, there is increasing recognition of the role of neuroinflammation as an initiation factor of neuron degeneration [50]. Recently, the innate immune receptors TLRs have been strongly linked to neurodegeneration [51]. In mammals there are at least 10 members of the TLR family that recognize specific components conserved among microorganisms. Activation of the TLRs promotes the production of inflammatory cytokines, creating an environment that could contribute to neuronal damage. It was recently suggested that TLRs have an important role in the crosstalk between neurons and glial cells in the central nervous system (CNS) [52].

By inducing the production of proinflammatory cytokines and cell adhesion molecules in immune cells, TLRs may indirectly damage neurons in conditions such as ischemic stroke and multiple sclerosis. Recent findings suggest that neurons also express a subset of TLRs and that their activation promotes neuronal degeneration in experimental models of stroke and Alzheimer's disease [51].

A great deal of experimental evidence points to role of TLRs in AD and multiple sclerosis, but very sparse information is available on the function of these receptors in other neurodegenerative disorders [51]. Moreover, a recent investigation on murine models of AD, PD, and ALS revealed dynamic changes in TLR expression [53]. In the mediator list associated to TLR pathway we identified the E3 ubiquitin ligase tumor necrosis factor—receptor-associated factor 6 (TRAF6). This member of TNF receptor-associated factor (TRAF) protein family has been associated to PD, HD, AD, and PD [54–56]. Thus, the present results support the hypothesis of an extensive involvement of TLRs in neurodegenerative disorders, pointing to the development of neuroprotective therapies by targeting these TLR-mediated inflammatory mechanisms, including cytokine-induced neurotoxicity.

The most represented connector protein is the growth factor receptor-bound protein 2 (GRB2), a widely expressed protein that is essential for multiple cellular functions. Inhibition of GRB2 function impairs developmental processes in various organisms and blocks transformation and proliferation of various cell types. A role for GRB2 in the pathogenesis of AD has been suggested [57]. GRB2 binds to phospho-A*β*PP (amyloid beta precursor protein) and it is in complex with A*β*PP in human brains. Both of these complexes are augmented in brains of Alzheimer's affected subjects. GRB2 is best known for its ability to link the epidermal growth factor receptor tyrosine kinase to the activation of Ras and its downstream kinases, ERK1,2 [58]. Experimental data suggest that chronic activation of ERK plays a role in the mechanisms that trigger neurodegeneration. Thus, this could advocate for a central role of this protein and the pathways in which it is involved (e.g., RAS) in the neurodegenerative process and it could be considered as a potential target for pharmacological intervention.

The pairs of diseases with the highest number of shared connector proteins are FTP-PD, ALS-PD, PD-HD, and FTD-ALS. Among these, we focused on the FTD-PD and ALS-PD connectors based on the differential “ranking” in the network versus the text mining approach of these disease pairs (i.e., high similarity according to network analysis, and low similarity in text mining in (additional file 1: Suppl.5)) suggesting the potential of the network approach to uncover the link between diseases that even with text mining approaches are unrelated.

### 4.3. FTD and PD Link

FTD and PD are neurodegenerative diseases with distinct clinical and pathological features, with FTD being a heterogeneous disorder characterized by behavior and language disturbances, associated with degeneration of the frontal and temporal lobes without major movement disturbances, while PD is characterized by substantia nigra dopamine cell degeneration and by the cardinal motor signs and symptoms including the resting tremor, rigidity, and akinesia.

The GO terms most strongly enriched among FTD-PD connector proteins are related to apoptosis (additional file 1: Suppl.6) underlining the centrality of this process in this disease pair and, in particular, to signaling by caspase family having caspase-1, -3, and -8 in the list. Activated caspase-1 (interleukin-1*β* converting enzyme) and caspase-3 (Yama/Apopain/Cpp32) cleave proteins that are important in maintaining cytoskeletal integrity and DNA repair and activate deoxyribonucleases, producing cell death with morphological features of apoptosis [59, 60]. There are various lines of evidence suggesting that caspases 1, 3 and 8 are implicated in diverse neurodegenerative diseases. Caspase-3-dependent proteolytic activation of protein kinase CD (PKCD) contributes to the degenerative process in dopaminergic neurons [61] and, in MPTP-induced Parkinson's disease in mice, gene disruption of caspases 1 and 3 prevents disease development. Moreover, in Parkinson's disease, Caspase-8 is an effector in apoptotic death of dopaminergic neurons [62].

In FTD, there is evidence of activated caspase-3 expression in neurons and astrocytes, which may contribute to neuronal cell death and astrocyte degeneration in the FTD brain [63]. These experimental data are then in line with our network-based approach, underlining the role of apoptosis and of caspase in the pathophysiology of PD and FTD diseases.

### 4.4. ALS and PD Link

ALS and PD are both movement disorder, but with specific clinical aspects and histopathological markers. In its classic form, ALS affects motor neurons at upper and lower levels leading to progressive muscle weakness and atrophy.

The ALS-PD connector proteins are overrepresented in GO terms related to response to growth factor stimulus (additional file 1: Suppl.6). The gene expression programs activated by these pathways initiate a spectrum of fundamental cellular activities including proliferation, growth (increase in cell size), differentiation, and survival [64, 65]. These processes are critical for normal embryonic development and adult homeostasis and are frequently aberrantly activated in cancer.

Of interest, a connector protein member of these pathways is glycogen synthase kinase 3 (GSK-3*β*), which is a proline-directed serine-threonine kinase that was initially identified as a phosphorylation and inactivation agent of glycogen synthase. GSK-3*β* sits at the convergence of several signaling pathways that are critical for neuronal viability and proper function and several apoptotic stimuli, including A*β* peptide, ischemia, and neurotoxins [66–68]. Increased activity of glycogen synthase kinase-3 (GSK-3) has recently been emphasized as an important pathogenic mechanism of neurodegenerative disease, including Alzheimer's disease and ALS [69]. ALS is associated with the elevated expression and/or activation of GSK-3 *β* [70] and GSK-3*β* has been suggested to have an activity in motor neuronal cell death [71]. In addition, several studies demonstrate the importance of this kinase in the genesis and maintenance of neurodegenerative changes associated with PD since it could interfere with two of the major degenerative processes associated with PD: tau hyperphosphorylation and *α*-syn-induced toxicity, due to increased accumulation of this protein [72]. Finally, compounds that inhibit GSK-3 such as lithium and valproate are able to delay the onset, reduce neurological deficits, and prolong survival in an ALS mouse model [73]. In addition, they have been proposed as therapy for PD since it can prevent both *α*-synuclein accumulation and neurodegeneration in an animal model of the disease [74]. Therefore, our data support GSK-3 as novel site of intervention in the treatment and management of these diseases.

## 5. Conclusions 

In conclusion, the present paper has proposed a network-based approach to efficiently infer the network of neurodegenerative diseases. The network mining showed advantages on both the construction of the disease network and the inference of molecular mechanisms underlying the linkage between diseases. This network-based approach offered the possibility to explore the molecular pathways involved in neurodegenerative diseases, identifying Toll-like receptors as a central molecular signaling pathway in neurodegeneration and providing potential candidate proteins to be targeted by pharmacological intervention (e.g., TRAF6). Moreover, it offered the possibility to investigate the direct and indirect relationship between apparently distinct diseases, suggesting common molecular alteration, including prion protein in HD and PD and GSK-3*β* in ALS and PD.

## Supplementary Material

Suppl 1: The list of disease proteins and their corresponding diseases.
Suppl 2: The lists of disease proteins occurring in the six corresponding components.
Suppl 3: The computations and distribution diagrams for network centrality indices. Each worksheet presents one centrality.
Suppl 4: The list of connector proteins of the different diseases.
Suppl 5: The ranking of disease association using the network-based approach and the text mining approach. The same disease pair in the different rows is highlighted by the same color.
Suppl 6: Network of significantly enriched GO terms.
This schematic network illustrates GO terms that were significantly enriched in the ALS-PD (A) and FTD-PD (B) connector proteins, as well as the overlap between related terms. GO terms containing at least 10 connector proteins, and occurring in levels 3-8 of the GO hierarchy were considered in the analysis. Terms that were significantly enriched (p<0.05 after correction for multiple testing) with connector proteins are depicted. Node colours and inset bar indicate the p value for enrichment of each term, after correction for multiple testing.

## Figures and Tables

**Table 1:** Statistics of six components in the ND PPI network.

Component	Nodes	Edges	Density	Min degree	Max degree	Avg degree
C1	1198	1502	0.002	1	133	2.508
C2	10	9	0.200	1	9	1.800
C3	3	2	0.667	1	2	1.333
C4	4	3	0.500	1	3	1.500
C5	5	4	0.400	1	4	1.600
C6	2	1	1.000	1	1	1.000

**Table 2:** The top 20 proteins ranked by four network indices.

Rank	Uniprot ID	*D*	Uniprot ID	*C*	Uniprot ID	*B*	Uniprot ID	TI^3^
1	P04626	133	P10636	0,000283	P04626	138806,9	P04626	223,9606
2	P20226	125	P62993	0,000277	P20226	124955,9	P20226	191,1601
3	P04156	83	P37840	0,000273	P04156	112084,5	P04156	140,9858
4	Q16637	82	P20226	0,000273	P37840	91931,3	Q16637	140,258
5	P55072	78	P04626	0,00027	Q16637	89120,94	P55072	121,1479
6	P37840	72	Q14203	0,000267	P55072	84506,5	P49768	101,3766
7	O60260	67	P62988	0,000267	P62993	83655,6	P42858	99,16651
8	P49768	67	P04156	0,000266	P10636	78425,93	P37840	96,52639
9	P42858	64	P42858	0,000266	P42858	77523,24	O60260	95,05239
10	P10636	58	P49768	0,000261	P49768	67333,14	P37231	88,60983
11	P37231	57	P09936	0,00026	O60260	62325,74	P01023	84,57448
12	Q14203	51	P55072	0,00026	P01023	62196,92	Q14203	82,81538
13	P35637	49	P35637	0,000258	P37231	58762,26	P10636	71,14661
14	P01023	47	P51587	0,000258	Q14203	55777,51	P35637	70,99781
15	P21675	44	O60260	0,000256	P51587	51505,59	P51587	64,43589
16	P51587	42	Q16637	0,000249	P62988	49875,5	P21675	52,16238
17	P22314	31	Q9UNE7	0,000247	P35637	46745,78	Q6Y7W6	46,97096
18	Q6Y7W6	30	P21675	0,000247	Q6Y7W6	38551,43	P43354	41,79592
19	P43354	28	P05067	0,000247	P05067	37130,13	P22314	41,70855
20	P09936	26	P37231	0,000247	P09936	29961,11	P28799	32,43995

The “gene symbol” column shows the corresponding genes of top 20 proteins (in UniprotID) ranked by degree values.

**Table 3:** Common proteins between NDs.

Disease	Uniprot ID	Gene symbol	Gene name
PD, HD	P20226	TBP	TATA box binding protein
HD, P	P04156	PRNP	Prion protein
FTD, PD	P37840	SNCA	Synuclein, alpha
	P10636	MAPT	Microtubule-associated protein tau
	Q96QT4	TRPM7	Transient receptor potential cation channel, subfamily M, member 7
	Q16143	SNCB	Synuclein, beta
	Q99497	PARK7	Parkinson disease (autosomal recessive, early onset) 7
FTD, ALS	Q96QT4	TRPM7	Transient receptor potential cation channel, subfamily M, member 7
	Q13148	TARDBP	TAR DNA binding protein
	Q99497	PARK7	Parkinson disease (autosomal recessive, early onset) 7
PD, LBD	P37840	SNCA	Synuclein, alpha (non-A4 component of amyloid precursor)
	Q16143	SNCB	Synuclein, beta
FTD, LBD	P37840	SNCA	Synuclein, alpha (non-A4 component of amyloid precursor)
	Q16143	SNCB	Synuclein, beta
ALS, PD	Q96QT4	TRPM7	Transient receptor potential cation channel, subfamily M, member 7
	Q99497	PARK7	Parkinson disease (autosomal recessive, early onset) 7
ALS, SMA	O95292	VAPB	VAMP (vesicle-associated membrane protein)

## List of Abbreviations

ND: Neurodegenerative disease
i2d: Interologous Interaction Database
OMIM: Online Mendelian Inheritance in Man
UniProt: The universal protein resource
*D*: Degree index
*C*: Closeness index
*B*: Betweenness index
TI^*n*^: Topological index at step *n*
HD: Huntington's disease
P: Prion
FTD: Frontotemporal dementia
AD: Alzheimer's disease
FA: Friedreich's ataxia
LBD: Lewy body disease
PD: Parkinson's disease
ALS: Amyotrophic lateral sclerosis
SMA: Spinal muscular atrophy
GBM: Glioblastoma multiforme.
