# Supplementary figures and images for: Network Analysis of Neurodegenerative Disease Highlights a Role of Toll-Like Receptor Signaling
Source: Biomed Res Int. 2014 Jan 16;2014:686505. doi: 10.1155/2014/686505 (PMC3914352; doi:10.1155/2014/686505)

A

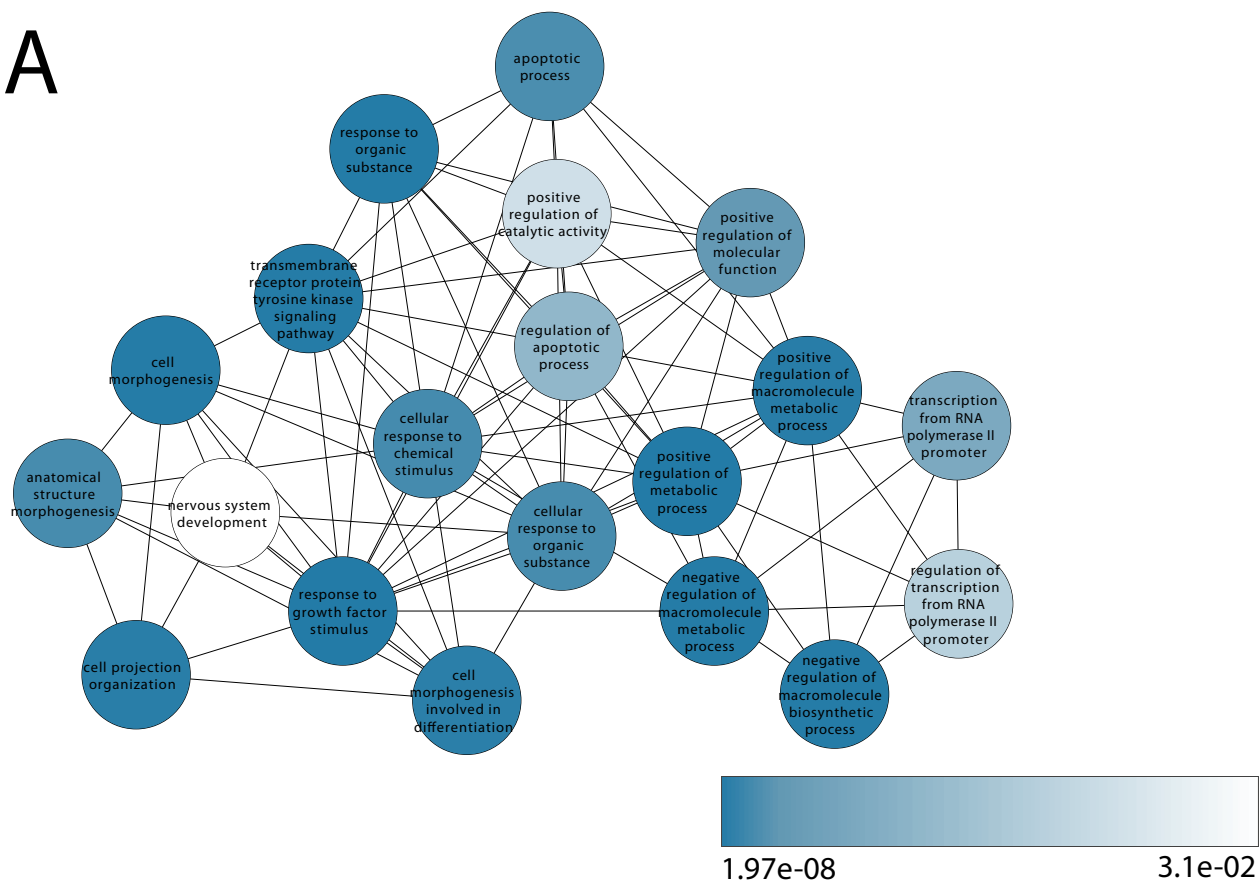

B

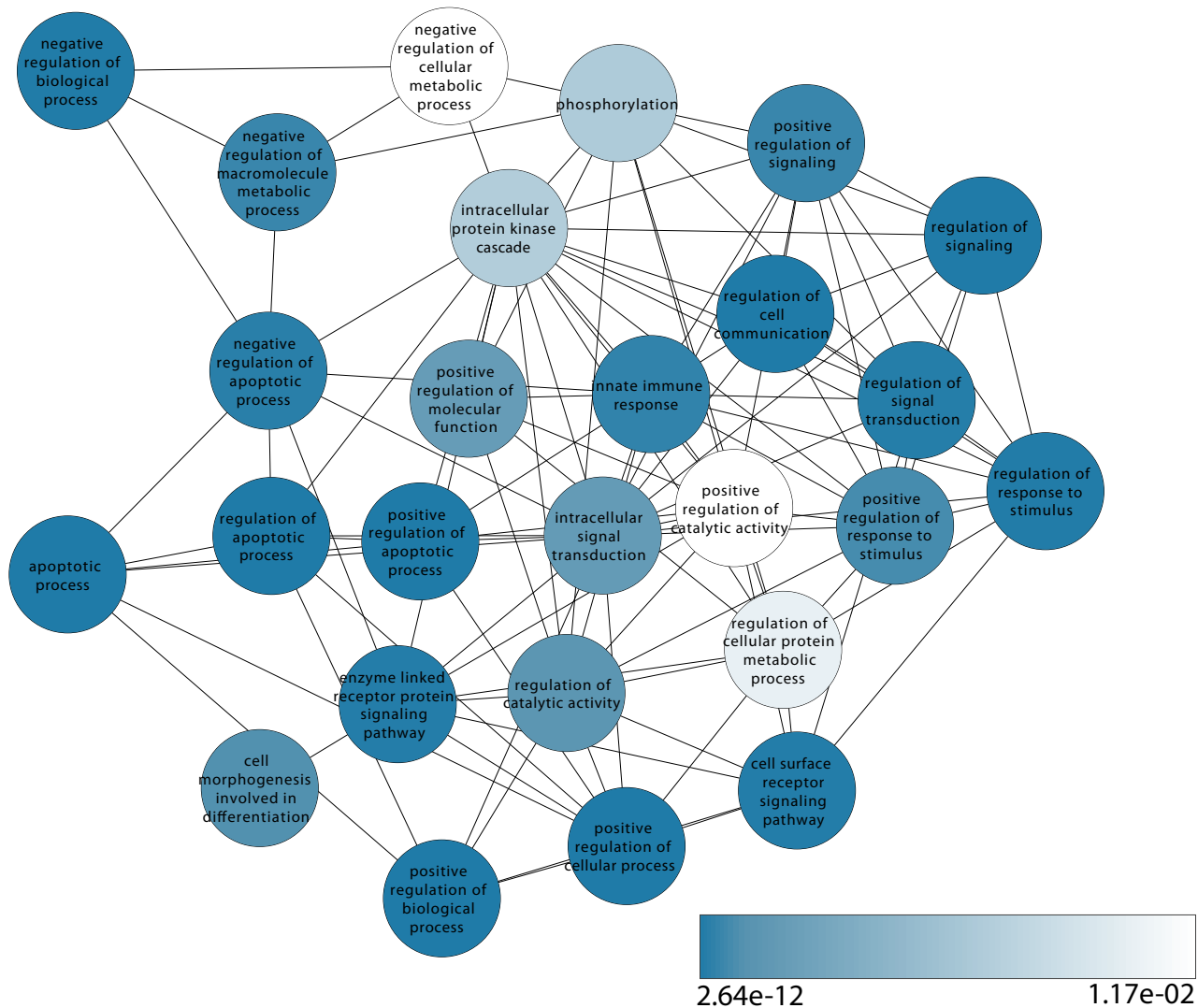

Supplement: Supplementary file 1 — Suppl 1: The list of disease proteins and their corresponding diseases. Suppl 2: The lists of disease proteins occurring in the six corresponding components. Suppl 3: The computations and distribution diagrams for network centrality indices. Each worksheet presents one centrality. Suppl 4: The list of connector proteins of the different diseases. Suppl 5: The ranking of disease association using the network-based approach and the text mining approach. The same disease pair in the different rows is highlighted by the same color. Suppl 6: Network of significantly enriched GO terms. This schematic network illustrates GO terms that were significantly enriched in the ALS-PD (A) and FTD-PD (B) connector proteins, as well as the overlap between related terms. GO terms containing at least 10 connector proteins, and occurring in levels 3-8 of the GO hierarchy were considered in the analysis. Terms that were significantly enriched (p<0.05 after correction for multiple testing) with connector proteins are depicted. Node colours and inset bar indicate the p value for enrichment of each term, after correction for multiple testing. [file 686505.f1.zip › Suppl6_GOTermAnalysisforConnectorProteinsALS_PDandFTD_PD.pdf]
